# The Puerto Rico Alzheimer Disease Initiative (PRADI): A Multisource Ascertainment Approach

**DOI:** 10.3389/fgene.2019.00538

**Published:** 2019-06-19

**Authors:** Briseida E. Feliciano-Astacio, Katrina Celis, Jairo Ramos, Farid Rajabli, Larry Deon Adams, Alejandra Rodriguez, Vanessa Rodriguez, Parker L. Bussies, Carolina Sierra, Patricia Manrique, Pedro R. Mena, Antonella Grana, Michael Prough, Kara L. Hamilton-Nelson, Nereida Feliciano, Angel Chinea, Heriberto Acosta, Jacob L. McCauley, Jeffery M. Vance, Gary W. Beecham, Margaret A. Pericak-Vance, Michael L. Cuccaro

**Affiliations:** ^1^Department of Internal Medicine, Universidad Central Del Caribe, Bayamón, PR, United States; ^2^John P. Hussman Institute for Human Genomics, University of Miami Miller School of Medicine, Miami, FL, United States; ^3^VA Caribbean Healthcare System, San Juan, PR, United States; ^4^Clínica de la Memoria, San Juan, PR, United States

**Keywords:** Alzheimer disease, ascertainment, PRADI, genetics, community resources, ADSP, diversity, health disparities

## Abstract

**Introduction:**

Puerto Ricans, the second largest Latino group in the continental US, are underrepresented in genomic studies of Alzheimer disease (AD). To increase representation of this group in genomic studies of AD, we developed a multisource ascertainment approach to enroll AD patients, and their family members living in Puerto Rico (PR) as part of the Alzheimer’s Disease Sequencing Project (ADSP), an international effort to advance broader personalized/precision medicine initiatives for AD across all populations.

**Methods:**

The Puerto Rico Alzheimer Disease Initiative (PRADI) multisource ascertainment approach was developed to recruit and enroll Puerto Rican adults aged 50 years and older for a genetic research study of AD, including individuals with cognitive decline (AD, mild cognitive impairment), their similarly, aged family members, and cognitively healthy unrelated individuals age 50 and up. Emphasizing identification and relationship building with key stakeholders, we conducted ascertainment across the island. In addition to reporting on PRADI ascertainment, we detail admixture analysis for our cohort by region, group differences in age of onset, cognitive level by region, and ascertainment source.

**Results:**

We report on 674 individuals who met standard eligibility criteria [282 AD-affected participants (42% of the sample), 115 individuals with mild cognitive impairment (MCI) (17% of the sample), and 277 cognitively healthy individuals (41% of the sample)]. There are 43 possible multiplex families (10 families with 4 or more AD-affected members and 3 families with 3 AD-affected members). Most individuals in our cohort were ascertained from the Metro, Bayamón, and Caguas health regions. Across health regions, we found differences in ancestral backgrounds, and select clinical traits.

**Discussion:**

The multisource ascertainment approach used in the PRADI study highlights the importance of enlisting a broad range of community resources and providers. Preliminary results provide important information about our cohort that will be useful as we move forward with ascertainment. We expect that results from the PRADI study will lead to a better understanding of genetic risk for AD among this population.

## Introduction

Alzheimer disease (AD) is a progressive neurodegenerative disorder that affects 1 in 9 Americans over the age of 65. This disease has a significant impact on individuals with AD and their families and poses huge financial and social burden on society. To date, over 20 loci have been identified as risk factors for AD in non-Hispanic White (NHW), genome wide association studies (GWAS) with limited GWAS in other populations (Lambert et al., 2013). In addition, the only large AD sequencing effort to date, the Alzheimer’s Disease sequencing project (ADSP) (Beecham et al., 2017), has focused its efforts on individuals of NHW descent, including a limited number of Hispanic (HI), and African American individuals. The importance of examining AD in other populations (Ramirez et al., 2008) is highlighted by findings that show Caribbean Hispanics from the Dominican Republic are twice as likely as NHW to have late-onset Alzheimer’s Disease (LOAD) (Tang et al., 1998, 2001). Furthermore, the incidence of new LOAD cases in families from the Dominican Republic is three times larger than the incidence found in NHW families (Vardarajan et al., 2014) even though the genetic risk of LOAD is similar. Despite clear evidence that points to the importance of investigating AD in underserved populations, this work has lagged.

Although comparisons of risk among different ethnic groups are complicated by differences in the assessment of cognitive decline across studies and population differences in willingness to participate in medical research, there are several possible explanations for increased incidence in these specific ethnic groups (e.g., lower educational attainment, higher rates of cardio- and cerebrovascular disease, and metabolic syndrome). While the importance of diversity and inclusion in genomic research has been emphasized for more than two decades (NIH Revitalization Act of 1993, Public law 103–143) many groups, including Hispanics, are underrepresented in biomedical research (Shavers et al., 2002; Sheppard et al., 2005; Calderon et al., 2006), including genomic, and translational studies (Armstrong et al., 2005; Ricker et al., 2006; Armstrong et al., 2012). Further, this lack of participation has the potential to delay the application of novel treatments that may be relevant to these populations, exacerbating existing health disparities in a variety of diseases, including AD. Specifically, given the importance of genomic research in the development and implementation of precision medicine initiatives (Hampel et al., 2017), there is an urgency to engage with and include underserved and underrepresented groups in such research to enable access to these advanced treatments (Wilkins, 2018).

Alzheimer disease is the most common form of dementia and the fourth leading cause of death in Puerto Rico (PR) (Friedman et al., 2016). The population of PR was estimated at 3,474,182 individuals in 2015, with 617,007 over the age of 65, and AD prevalence of 12.5% (Puerto Rico Department of Health, 2015). Further, according to Perreira et al. (2017) the population of PR is aging and struggles with high rates of comorbid conditions (e.g., hypertension and diabetes) that contribute to dementia. These numbers underscore the need to investigate early risk factors and develop the necessary research to study the neurobiology of cognitive decline in Puerto Ricans and more broadly Hispanics. Furthermore, enriching AD genomic studies with Hispanic populations is fundamental for reducing health disparities, delivering precision medicine, and ultimately improving health outcomes for this community.

To address the range of disparities experienced by Hispanics due to under-representation in genomic studies of AD, we developed the Puerto Rico Alzheimer Disease Initiative (PRADI). The goal of this National Institute of Aging funded project is twofold. First, the PRADI study examines genomic risk for AD in Puerto Ricans and adds to the growing body of knowledge regarding Hispanic risk for AD. Second, the PRADI study makes comparisons using two types of controls: family-based (related controls) and case-control (unrelated controls), paralleling, and building on the ongoing work of the ADSP (Beecham et al., 2017). Furthermore, Puerto Ricans are an admixed population, enriched for at least three ancestries (European Caucasian, Western African, and Amerindian/Taino), resulting in complex population substructure (Claudio-Campos et al., 2015; Rajabli et al., 2018). The use of population substructure (i.e., global and local ancestry) can allow for adjustment of models to improve genetic analyses. The importance of examining ancestral contributions in Hispanics can be seen in studies of complex diseases, including asthma (Gignoux et al., 2019), multiple sclerosis (Amezcua et al., 2018), and cancer (Salgado-Montilla et al., 2017; Diaz-Zabala et al., 2018). The usefulness of understanding and incorporating genotypic and admixture information into the conceptualization and management of disease among Puerto Ricans is becoming increasingly apparent (Morales-Borges, 2017; Diaz-Zabala et al., 2018).

In contrast to other studies of Puerto Ricans (Tucker et al., 2010), the current study focuses exclusively on participants from the island of PR. We describe the design and implementation of our multisource method for recruiting individuals for the genetic study of AD and our corresponding work in the community to increase study participation among eligible Puerto Ricans. Equally important, we describe our cohort with respect to clinical features and ancestral proportions by region. These results provide a preliminary picture of our PRADI cohort.

## Materials and Methods

A multisource ascertainment approach was implemented to recruit and enroll participants into the PRADI study. As described below, the approach consisted of different phases that revolved around community engagement and included: (a) identification and relationship building with key stakeholders from several organizations; (b) collaborative agreement on ascertainment methods and formalization using memorandums of understanding; (c) targeted actions and recruitment events; and (d) education and dissemination of information about AD to health professionals and the general public. This approach was designed to establish and strengthen collaborative relationships with key stakeholders to facilitate ascertainment for this study and future studies.

Ascertainment efforts were carried out in PR and encompassed all seven health regions (Arecibo, Bayamón, Caguas, Fajardo, Mayagüez, Metro, and Ponce) as defined by the Puerto Rico Department of Health. Only bilingual personnel were sent to the sites and plain Spanish was used for all verbal and written study-related communication (materials for public dissemination were developed for a third-grade reading level). Standard screening and evaluation activities were performed, which included collection of clinical, family, and medical history and neurocognitive testing. Individuals were determined to be cases or controls with further specification depending on whether they were family history positive or negative for AD.

Finally, to investigate potential differences among our participants from different parts of the island, we tested for differences in age of onset and 3MS scores by health region and ascertainment source (i.e., AD specialist, adult care center, or community event/activity). We also conducted admixture analysis to examine the population substructure of our Puerto Rican cohort by region to evaluate differences in ancestry proportions among the health regions.

### Ascertainment Procedures

#### Ascertainment Phase One: Getting to Know the Field Stakeholders From Multiple Sectors

**FIGURE 1:**
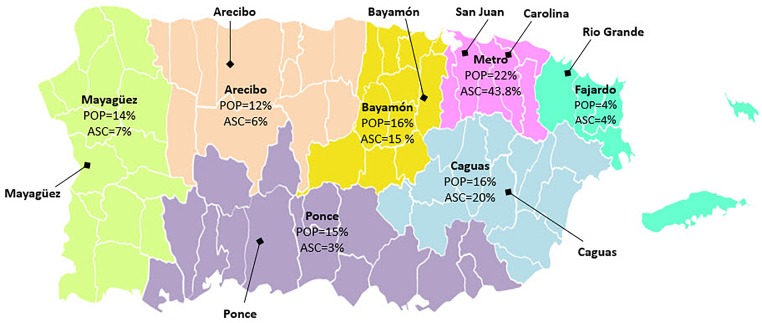
Percentages of PRADI Ascertainment (*N* = 674) by Puerto Rico Department of Health Regions.

In the initial phase of our multisource ascertainment approach, the local team identified potential sources of participants within PR communities by interacting with groups and providers that serve the AD population. There are multiple groups and ongoing community initiatives working to increase AD awareness in PR. Our goal was to establish collaborative relationships with stakeholders from different sectors (Figure 1). These interactions served as a starting point to disseminate information about the study, to identify sources for cases and controls, to build networks with potential collaborators, and to create opportunities for direct ascertainment. In addition, these initial meetings served as a venue for discussing the importance of inclusive recruitment in genetics research, especially how a lack of diversity can delay specific populations’ access to personalized/precision medicine. The primary groups we approached included:

#### Governmental Stakeholders

We contacted central and local government representatives, including the PR office of the Ombudsman for the Elderly (OPPEA, for its Spanish acronym), a legal affairs office for older adults, and the AD Registry of the Health Department of PR. As an initial step, local team members joined the Health Department Alzheimer’s Advisory Board. This process allowed us to meet with key stakeholders to discuss the PRADI study. Through these initial contacts, OPPEA provided us with additional contacts at the provider level to include various programs and adult care centers for older adults and those with AD and other cognitive problems. Through these contacts, we established ties with additional local government representatives of the municipalities, including Cidra, Fajardo, Carolina, Aguadilla, Arecibo, among others.

#### Community Non-profit Organizations (NPO)

To establish community based collaborations in the non-profit sector, we contacted multiple groups that serve older adults in PR, including the Puerto Rican Chapter of the AARP; Mente Activa (Active Mind), which is a non-profit organization that promotes physical and mental activity for older adults and those with dementia; and Organización Pro Ayuda a Personas con Alzheimer (OPAPA), another non-profit organization that provides education and support to people with AD and their families. Our team met with leadership in these organizations to provide information about the PRADI study.

#### Religious Groups

Our primary religious contact was the Lutherans Social Service of PR, a non-profit faith-based organization involved in providing services to older adults. It is funded to provide programs to train dementia capable personnel and service providers as well as programs to identify older adults with early signs of AD. In addition, we contacted the Catholic Church, especially the Seminary of PR and the Caguas Cathedral. Both groups agreed to assist with the study by providing access to participants and disseminating information about our study during religious services and through print media.

#### Ascertainment Phase Two: Defining and Formalizing Collaborations With Stakeholders

The next phase in our multisource ascertainment approach was seeking and using input from the stakeholders and organizations about best practices for ascertainment. This process typically involved in person discussions between the local team (headed by Dr. Feliciano, a neurologist who specializes in the care of older individuals) and the organizations. This allowed us to define our ascertainment practices in alignment with accepted practices for the respective organizations, groups, etc. In addition, it allowed us to address any concerns at the outset. Based on these discussions, we constructed memorandums of understanding (MOUs) to specify the nature of the relationship and outline collaborative activities with the stakeholders from different sectors. MOUs were signed with OPPEA, the Puerto Rican Chapter of the AARP and Lutheran Social Services of PR. In addition, we established MOUs with Mayors and their staff from several municipalities, including Cidra, Fajardo, Carolina, Aguadilla, and Arecibo. As part of the MOU, the Universidad Central del Caribe provided insurance endorsements for the use of their venues during recruitment events.

#### Ascertainment Phase Three: Targeted Actions and Direct Recruitment

Working with the various groups with whom we had MOUs, we set up multiple recruitment events. Depending on the site, pre-recruitment conferences were scheduled to educate center personnel (e.g., primary doctors, nurses, social workers, psychologist, and others dementia specialists) or the public. These pre-recruitment meetings were used to provide general information about AD and to clarify aspects of the study in person to healthcare providers as well as potential participants and their families. At meetings involving the public, potential participants, or family members we gathered contact information for further follow up, leading to recruitment of interested individuals. This also allowed us to estimate the number of participants and to plan our ascertainment resources accordingly.

#### Ascertainment Phase Four: Giving Back: Dissemination and Education

We conducted a number of follow-up events to provide information for caregivers and center personnel at the various recruitment sites. For physicians, we were able to provide continuing medical education through the Puerto Rican College of Physicians and Surgeons; for health professional staff, we provided participation certificates for early detection of AD and culturally relevant adaptation of the comprehensive and evidence-based community support strategies.

This follow-up allowed us to disseminate information about AD to the community. The provision of information about AD to non-AD healthcare workers and general communities will help us build local resource networks and empower them with knowledge about dementia capabilities to improve the quality of life of the participants and their caregivers. In addition, at select venues we have also organized educational outreach activities where we served as expert speakers, providing information about dementia research and care. Typical audiences included healthcare providers (e.g., nurses, social workers, case managers, and primary care physicians) and the public. We have also engaged in dementia-related initiatives via social media, like “Un café por el Alzheimer” (A cup of coffee for Alzheimer) (Friedman et al., 2016), which shares our study information on their social media platforms.

### Study Population

A convenience sampling method with a geographic distribution throughout the island was used. PRADI participants were self-reported Puerto Rican adults, aged 50 years, and older with no restrictions on gender or socioeconomic status. While the majority of participants were residents of PR, a small fraction of relatives of the Puerto Rican families living in the continental United States (Florida, New York, Connecticut, and Massachusetts) were enrolled. In addition, some individuals less than 50 years of age were enrolled. When conducting our analyses, we included only residents of PR who were 50 years of age or greater.

Our cohort is further specified based on seven health regions as defined by the PR Department of Health^1^. These seven regions contain multiple municipalities and place this cohort in the context of the previously established health related structure. Each of the health regions is labeled by the major municipality within each region (with the exception of the Metro region). As seen in Figure 1, the most heavily populated areas per the 2010 census are the Metro, Caguas, and Bayamón regions, containing 22, 16, and 16% of the total population, respectively. Per the same census period, ∼15% of individuals in PR were over 65 years of age.

### Ascertainment Sources

All participants were ascertained via three main sources: AD specialists, adult care centers, and community events. This approach allowed us to capture a wide range of AD cases from varied socioeconomic backgrounds and education levels. All individuals were recruited using site-specific IRB approved protocols.

#### AD Specialists

Several AD specialists (neurologists, psychiatrists, and geriatricians) served as collaborators and referred patients who met inclusion criteria and were interested in participating in the PRADI study. These included patients with AD, mild cognitive impairment (MCI), and dementia. As described below in the screening and evaluation section, we obtained clinical and medical records for patients who were recruited via AD specialists.

#### AD Centers and Adult Care Centers

To date, we have recruited participants from seven AD dedicated centers and advanced age nursing homes across the island, identified through the OPPEA directory of services website. The AD centers and nursing homes serve between 20 and 40 individuals who are typically older than 60 years of age (with or without the diagnosis of AD) on a daily basis. These centers focus on providing therapeutic, social, and recreational activities to improve quality of life, as well as educating, and supporting caregivers or family members.

#### Community Groups

We conducted recruitment events in various municipalities. Typically, these recruitment events were preceded by a pre-recruitment event. The actual recruitment visits were then conducted at various centers or in private spaces. During these events, our multi-disciplinary teams consented participants (or their proxies), conducted cognitive screenings, and drew blood samples. These events ranged in size from small venues that attracted 20 or so individuals to much larger events that drew 60 or more individuals. We were able to enroll cases and controls during these events.

### Inclusion/Exclusion Criteria

Participants were enrolled in the following categories: cases (AD and MCI), unaffected family members of cases, or unrelated individuals with no cognitive problems. To be enrolled, participants had to meet basic inclusion criteria. All individuals had to: (a) be of Puerto Rican ancestry (with at least one grant-parent born on the island); (b) be ≥50 years of age; and (c) be willing to participate (or, in cases of serious cognitive impairment, have family members who consent on their behalf) and provide informed consent or have a proxy for consent.

To be included as a case, we required that individuals have a previous clinical diagnosis of AD, MCI, dementia, or show evidence of a memory disorder, and meet standard criteria for AD or MCI (McKhann et al., 1984; Albert et al., 2011; McKhann et al., 2011). We included cases from families (family history positive) as well as sporadic or isolated cases (family history negative). We excluded individuals whose memory and cognitive problems are secondary to other causes (e.g., stroke, psychoses, etc.) and those with a known mutation (e.g., PS1, PS2, or APP).

To be included as a control, individuals had to meet basic inclusion criteria, have no prior clinical diagnoses of a memory disorder or subjective memory complaints, demonstrate no cognitive problems on neurocognitive screening and assessment, and be unrelated to our cases. Unaffected family members had to meet the same inclusion criteria as the controls in addition to being a first- or second-degree relative of a case. For unaffected family members, we typically included the oldest available individual.

### Screening and Evaluation

For participants enrolled as cases (i.e., with suspected memory problems or known dementias), we conducted a detailed chart review during which we corroborated clinical diagnoses and extracted current and past medical histories, current and past medications, family histories (pedigrees), and sociodemographic information. In addition, we collected clinical neurologic and neuropsychological test data, neuroimaging results, and pertinent lab values (e.g., hematology, thyroid function, lipid profile, vitamin D and B12 levels, and liver function tests, hypothyroidism, and vitamin deficiency).

For presumptive cases, we conducted an initial screening with the Modified Mini-Mental State Examination (3MS) (Folstein et al., 1975; Teng and Chui, 1987) followed by a cognitive evaluation that included the NIA-LOAD cognitive battery (Morris et al., 2006; Weintraub et al., 2009). In addition, we administered the Clinical Dementia Rating Scale (CDR) (Yesavage, 1988). Individuals who were deemed cognitively normal were screened with the 3MS (Folstein et al., 1975; Teng and Chui, 1987) and the CDR. For most cognitively normal individuals, we administered the NIA-LOAD battery.

### Adjudication

All clinical, historical and screening/evaluation test data (e.g., laboratory tests, neurologic examination, neuroimaging, and neuropsychological screen and testing) from individuals with a known or suspected dementia were reviewed by a clinical adjudication panel consisting of a neurologist, neuropsychologist, and clinical staff. The panel reviewed all data and assigned best-estimate diagnoses. To be classified as AD individuals had to meet the current NIA-AA criteria (McKhann et al., 2011). They were further classified as definite (neuropathologic confirmation), probable, or possible AD. Diagnoses of MCI were assigned using the NIA-AA criteria (Albert et al., 2011). Cognitively normal individuals with no history of memory problems and MMSE or 3MS scores that fall above clinical cutoffs were designated as unrelated controls for the study. Family-based controls were evaluated similarly for inclusion in family-based analyses (Beecham et al., 2017). In the course of adjudication meetings, team members discussed cases until a diagnostic classification was determined. For those cases in which the team was unable to arrive at a final decision, the team stipulated the reason and corrective actions were taken (e.g., obtaining a more detailed history, retesting, etc.) In the event of a disagreement, the team consulted with an independent dementia specialist.

### Analysis

To test for possible differences in our cohort related to where participants live and how they were ascertained, we compared mean 3MS scores and mean age of onset (AAO) for cases by region and recruiting source. Cases consisted of both AD and MCI phenotypes. In addition, for our controls we were able to compare mean 3MS scores by region. All analyses were performed using one-way ANOVA in SAS and SPSS (SAS Institute Inc., 2011; SPSS, 2013). *P* values lower than 0.05 were considered statistically significant.

In addition, we conducted an admixture analysis to estimate the proportions of admixture (European, African, and Native American) in our cohort. Genotyping and quality control methods are described elsewhere (Alexander et al., 2009; Rajabli et al., 2018). Briefly, genotyping was performed on the Expanded Multi-Ethnic Genotyping Array and Global Screening Array (Illumina, San Diego, CA, United States) and quality was assessed using PLINK software, v.2. Using the reference panels (African, European, and Native American populations) from the Human Genome Diversity Project3, we conducted admixture analysis, using ADMIXTURE software (Alexander et al., 2009; Rajabli et al., 2018), to generate average ancestry proportions across PR’s seven health regions.

## Results

We have enrolled 770 individuals over a 30-month period, 710 of which were from PR. After removing individuals <50 years of age (35 unaffected, 1 MCI), our current dataset consisted of 674 individuals. The distribution of enrollment across the seven health regions of PR, as seen in Figure 1, shows the heaviest ascertainment in the Metro (44%; *N* = 295), Caguas (20%; *N* = 134), and Bayamón (16%; *N* = 106) regions, which reflects the greater population densities of these regions and cities. Enrollment numbers for the seven health regions are presented in Table 1, which also provides the numbers for the respective municipalities within those health regions.

**Table 1:** Ascertainment by health regions and municipalities (*N* = 674).

	Ponce	Arecibo	Mayagüez	Metro	Bayamón	Caguas	Fajardo
Population (%)	565,683 (15%)	456,036 (12%)	535,488 (14%)	822,562 (22%)	620,110 (16%)	589,795 (16%)	136,115 (4%)
Ascertainment (%)	23 (3%)	41 (6%)	49 (7%)	295 (44%)	106 (16%)	134 (20%)	26 (4%)
Ascertainment by municipalities (*n*)	Ponce (*n* = 15)	Arecibo (*n* = 15)	Aguadilla (*n* = 31)	San Juan (*n* = 157)	Bayamón (*n* = 33)	Cidra (*n* = 45)	Fajardo (*n* = 15)
	Juana Díaz (*n* = 3)	Manatí (*n* = 10)	Mayagüez (*n* = 7)	Carolina (*n* = 98)	Naranjito (*n* = 12)	Cayey (*n* = 27)	Luquillo (*n* = 6)
	Yauco (*n* = 3)		San Germán (*n* = 3)	Guaynabo (*n* = 28)	Orocovis (*n* = 12)	Caguas (*n* = 16)	Ceiba (*n* = 2)
	Adjuntas (*n* = 1)		Lajas (*n* = 3)	Trujillo Alto (*n* = 11)	Toa Alta (*n* = 12)	Naguabo (*n* = 16)	Rio Grande (*n* = 2)
	Guayama (*n* = 1)		Hormigueros (*n* = 2)	Canovanas (*n* = 1)	Toa Baja (*n* = 12)	Humacao (*n* = 8)	Vieques (*n* = 1)
			Isabela (*n* = 2) San Sebastián (*n* = 1)	Loíza (*n* = 1) Rio Piedras (*n* = 1)		San Lorenzo (*n* = 6) Gurabo (*n* = 4) Juncos (*n* = 4) Yabucoa (*n* = 4) Las Piedras (*n* = 2) Maunabo (*n* = 2)	


Among these 674 individuals, 282 (42%) were ascertained as AD, 115 (17%) were ascertained as MCI, and the remaining 277 (41%) were ascertained as unaffected. The majority of our cases (83%) had an age of onset ≥65 years of age. The greatest numbers of AD (*N* = 111; 39%) and MCI (*N* = 61; 53%) were ascertained in the Metro region. Equally high ascertainment numbers were also observed in Bayamón and Caguas (AD *N* = 54, 19%; MCI *N* = 17, 15%).

**FIGURE 2:**
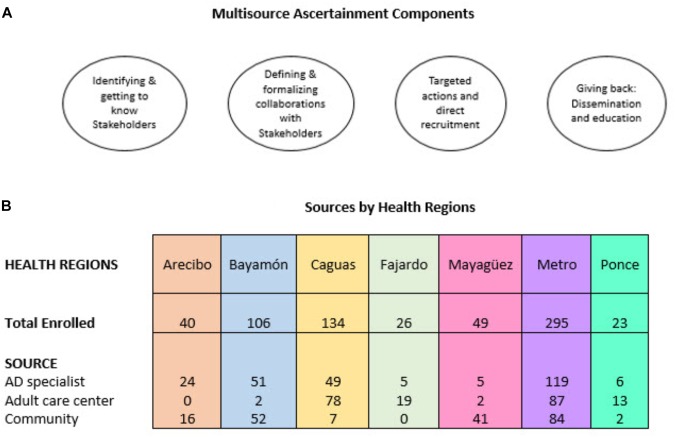
**(A)** Main components of multisource ascertainment approach and **(B)** resulting enrollment of study eligible individuals per health regions and by source within the health regions.

Participants were recruited from three sources: AD specialists (*N* = 261, 39%), adult care centers (*N* = 201, 30%), and community events (*N* = 202, 30%). Not surprisingly, as seen in Table 2, most of the AD cases were recruited via the AD specialist, while the largest number of MCI cases were ascertained through community events. Figure 2 provides additional information regarding enrollment sources per the respective health regions.

**Table 2:** Ascertainment by source (*N* = 664)^∗^.

	AD specialist	Adult care center	Community	Total
AD *N*	136	86	55	277
%	(49%)	(31%)	(20%)	

MCI *N*	43	17	53	113
%	(38%)	(15%)	(47%)	

UNAFF *N*	82	98	94	274
%	(30%)	(36%)	(34%)	

Total	261	201	202	664


*^∗^Our total ascertainment=674; 10 individuals were missing source data, A = Alzheimer disease; MCI = Mild cognitive impairment; UNAFF = Unaffected.*

Finally, our cohort can be further delineated by whether individuals were part of a family or ascertained as an isolated/sporadic case. Of the 43 multiplex families that have been completed to date, 10 families contain four or more living individuals with AD, 3 families contain 3 living individuals with AD, and 31 families contain 2 living individuals with AD. The mean number of LOAD cases per multiplex family is 3.9. Among the 198 individuals from those multiplex families 73 (37%) meet the criteria for LOAD, 19 (9%) meet the criteria for EOAD, 31 (16%) meet the criteria for MCI, and 75 (38%) meet the criteria for no cognitive problems.

### Admixture Results

**FIGURE 3:**
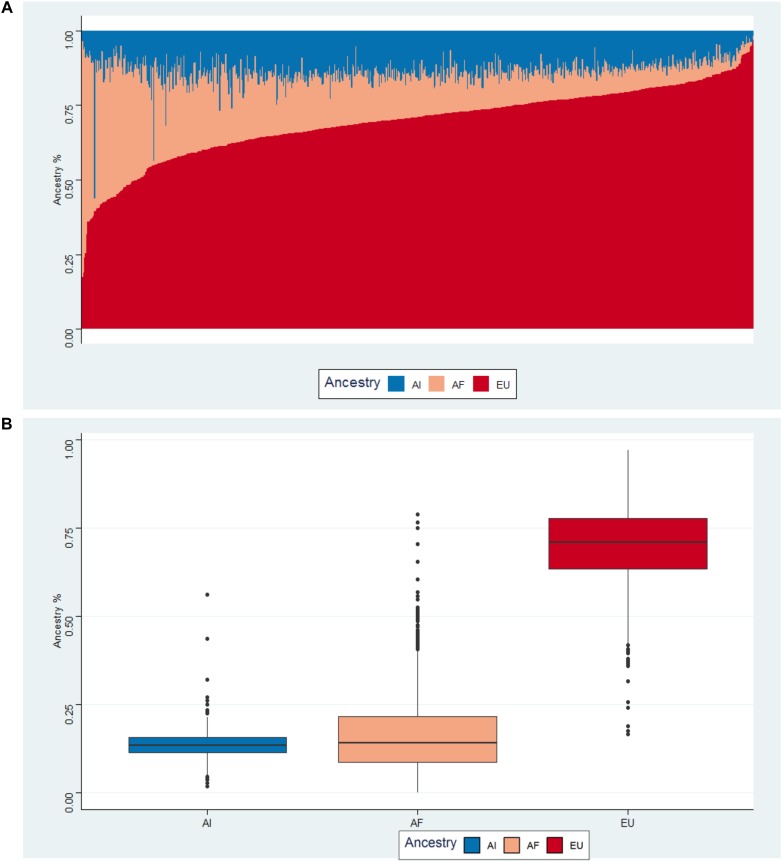
**(A)** Bar-plot of three way admixed Puerto Rican individuals estimated using ADMIXTURE software at *K* = 3. **(B)** Box-plot of average ancestries by health region.

We examined the population structure of Puerto Ricans using the supervised ADMIXTURE analysis at *K* = 3. Figure 3A illustrates the results from the ADMIXTURE analysis in a bar-plot figure. Each vertical bar represents an individual and corresponding estimates of the fraction of continental ancestries (African, European, and Native American). On average, Puerto Ricans have mostly European ancestry with a mean value of 69.3% (*SD* = 12.2). Mean values for African and Native American ancestry are 17.3% (*SD* = 12.2) and 13.4% (*SD* = 4.2), respectively as seen in the box plots (Figure 3B).

**FIGURE 4:**
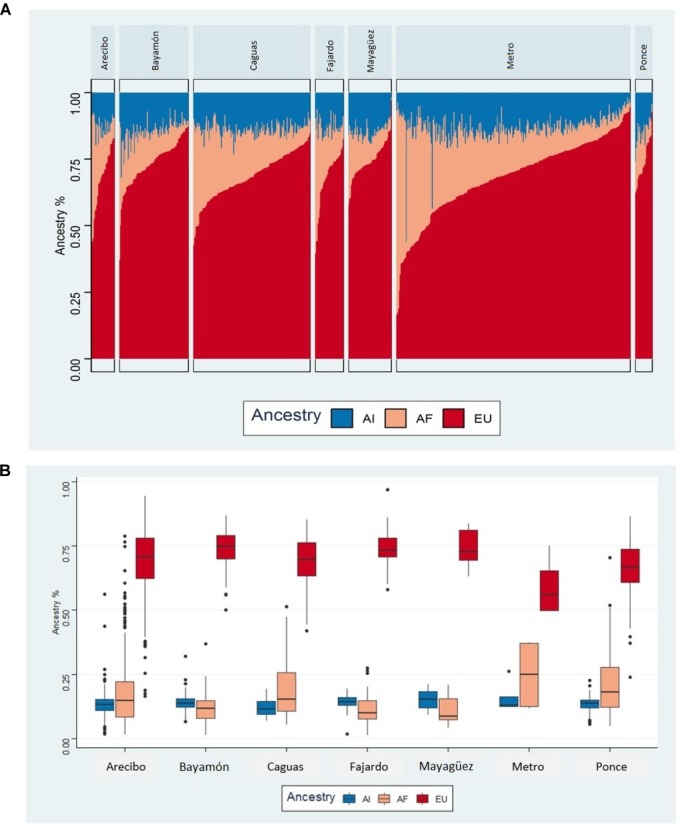
**(A)** Bar-plot of three way admixed Puerto Rican individuals for each of the health regions estimated using ADMIXTURE software at *K* = 3. **(B)** Box-plot of average ancestries by health region.

Figure 4A illustrates the bar-plots of admixed individuals across the Puerto Rican health zones and shows heterogeneous admixture patterns. Results of the admixture analysis are in general agreement with recent genetic studies showing a three-way admixture (European, African, and Native American) structure in Puerto Ricans (Via et al., 2011).

We observed a non-uniform distribution of European and African ancestral backgrounds across the health regions with relatively high European and low African ancestral proportions in Mayagüez, Ponce, and Bayamón (Figure 4B). The average European and African ancestry fractions in these zones are 74.4% (*s* = 5.8), 74% (*SD* = 8.1), 73.3% (*SD* = 8.7) and 11.9% (*SD* = 6.6), 11.6% (*SD* = 5.9), 11.0% (*SD* = 4.9), respectively. In contrast, the Native American ancestral background shows nearly uniform distribution across the geographical zones (Figure 4B).

### Clinical Comparisons

Separate one-way ANOVAs were conducted to test if mean values for AAO and the 3MS differed by (a) ascertainment region (i.e., the seven health regions of PR) and (b) ascertainment source (AD specialist, adult care center, and community).

#### Age at Onset (AAO)

The mean AAO values for our AD and MCI case were 74.1 (*SD* = 9.4) and 71.2 (*SD* = 8.5), respectively. As noted above, for the purposes of analysis we combined these into one group (cases) which had a mean AAO value of 73.2 (*SD* = 9.2). The mean values for AAO for the seven ascertainment regions and sources are shown in Table 3.

**Table 3:** Mean age at onset (AAO) and 3MS values for cases (*N* = 397) by region and source (note sample sizes reflect missing values).

	Age at onset		3MS	
		
REGION	*N*	Mean (*SD*)	*N*	Mean (*SD*)
Arecibo	27	71.6 (8.1)	17	56.7 (27.4)
Bayamón	69	73.1 (9.7)	36	62.9 (19.8)
Caguas	69	74.5 (9.9)	35	63.3 (28.8)
Fajardo	15	75.9 (9.6)	3^∗^	70.0 (10.1)^∗^
Mayagüez	22	70.3 (7.4)	19	46.3 (28.0)
Metro	172	73.1 (9.0)	101	69.5 (19.9)
Ponce	18	73.4 (9.7)	14	51.5 (24.6)
TOTAL	392	73.2 (9.2)	225	63.4 (68.4)

**SOURCE**	***N***	**Mean (*SD*)**	***N***	**Mean (*SD*)**

AD specialist	176	70.5 (9.0)	79	64.2 (27.6)
Adult care center	102	76.5 (9.0)	48	63.3 (18.9)
Community	107	74.6 (7.9)	92	63.1 (23.3)
TOTAL	385	73.2 (9.3)	219	63.5 (23.0)


*^∗^Removed from analysis of 3MS score by region.*

Across the different regions, mean AAO values ranged from 70.3 (*SD* = 7.4) in Mayagüez to 75.9 (*SD* = 9.6) in Fajardo. Results of one-way ANOVA found no statistically significant differences in AAO across the different health regions *F*(6,385) = 0.92, *p* = 0.48. The mean AAO values for the three ascertainment sources ranged from 70.6 (*SD* = 4.6) for AD specialists to 76.4 (*SD* = 9.0) for cases ascertained through adult care centers. The results of the one-way ANOVA found significant group differences among the three ascertainment sources *F*(2,382) = 16.29 *p* < 0.001. *Post hoc* tests showed mean AAO was higher in patients recruited from the community sites (+4.1 years) and adult care centers (+6.0 years) than it was for patients ascertained from AD specialists.

#### Modified Mini Mental State Examination (3MS)

The mean 3MS scores for our AD and MCI cases were 52.6 (*SD* = 23.5) and 80.1 (*SD* = 12.2), respectively; the overall mean 3MS score for all cases was 63.5 (*SD* = 24). The mean 3MS scores for the seven ascertainment regions and sources are seen in Table 3.

Among the health regions, mean 3MS scores ranged from 46.3 (*SD* = 28) in Mayagüez to 69.5 in the Metro region (*SD* = 19.9). Note that we dropped the Fajardo region, as there were only three 3MS scores. For these comparisons, the homogeneity of variances assumption was violated, as assessed by Levene’s Test of Homogeneity of Variance (*p* = 0.008). The one-way Welch ANOVA results show statistically significantly differences in mean 3MS scores between the health regions Welch’s *F*(5,52.96) = 3.81, *p* = 0.005. Games-Howell *post hoc* analysis revealed only one statistically significant comparison (*p* < 0.01) between the Metro and Mayagüez regions (23.3+5.8) [mean ± standard error]. For source, the mean values ranged from 63.1 (*SD* = 24) for cases ascertained via the community to 64.2 (*SD* = 27.6) for cases ascertained through AD specialists. Again, Levene’s Test of Homogeneity of Variance was significant (*p* = 0.03) indicating that the homogeneity of variances assumption was violated, prompting use of Welch’s ANOVA. Results of one way ANOVA found no statistically significant differences in 3MS means Welch’s *F*(2,130.5) = 0.04, *p* = 0.96.

## Discussion

Using a multisource approach that emphasized community engagement and was tailored to the Puerto Rican population, we were able to enroll eligible participants and their family members across PR. A major feature of our community engagement efforts was the development of partnerships with leaders of health initiatives in municipalities and resources within those municipalities. These included the health department, governmental organizations, community-based organizations, religious groups, and various healthcare providers. Establishing strong community partnerships allowed us to develop strategies with input from different parts of the community to achieve an ascertainment approach that was sensitive to the local culture.

Our multisource approach emphasizes community engagement beginning with the identification of and establishment of relationships with key stakeholder groups and organizations. This allowed us to develop mutually agreed upon ways to implement research activities and create memorandums of understanding to formalize implementation. Working with these stakeholders and organizations enabled us to conduct outreach and ascertainment activities in the respective municipalities. Concurrent with the outreach activities and recruiting events (and as a way of giving back to the communities), we provided information and educational opportunities to healthcare providers and the public. This community engagement approach, developed for PRADI by AD clinicians and researchers in Puerto Rico and Miami, is a platform for our ongoing ascertainment efforts.

Using this approach, we have enrolled 674 individuals from PR over the age of 50 for our PRADI study. These individuals were recruited fairly evenly from the three ascertainment sources are and concentrated in the three health regions with the largest numbers of individuals – Metro, Bayamón, and Caguas. We also observed that the main ascertainment sources varied by the health regions, reflecting different resources in the respective regions. Further, while the percentage of individuals ascertained in select regions paralleled the percentage of the total population for the region, the Metro and Ponce regions were disparate as 44% of our participants were ascertained in the Metro region which constitutes 22% of the population vs. 3% of our participants were ascertained in the Ponce region which constitutes 14% of the population. These ascertainment figures have already begun to inform our subsequent recruitment efforts, as we emphasized the need to engage other sectors of PR (e.g., Ponce).

The importance of recruiting in regions such as Ponce and Mayagüez is also reflected in the results of our admixture analysis showing differences in the proportion of European and African ancestry among individuals from these regions. The failure to ascertain participants from regions with different ancestral backgrounds could potentially limit the applicability of important findings to these groups. The significance of this for the PRADI study is reinforced by work showing that different ancestral backgrounds may play a significant role in modifying the effect of APOE on risk for AD (Rajabli et al., 2018). These results are preliminary and will need further investigation, in particular to specify area of origin for participants vs. current area.

In addition to potential ancestral differences across the different regions, we observed clinical differences in our cohort in relation to ascertainment region and sources. For instance, participants’ mean 3MS scores varied by ascertainment region although the only significant difference was between the Mayaguez and Metro regions. This may reflect differences in the sources of these participants as most of the individuals from Mayaguez were ascertained in the community. While there were no significant differences in AAO among participants from these different regions, we observed that AAO varied according to ascertainment source. Specifically, individuals who had been seen by AD specialists were more likely to have been identified as having cognitive/memory problems at younger ages. Aside from differences in sample size, the observed differences in AAO and 3MS values by ascertainment region and source most likely reflect the complex interplay of multiple influences, including access to AD specialists, availability of dementia related resources, and general knowledge and acceptance of AD.

The influence of knowledge and acceptance of AD is an important issue that is intertwined with efforts to recruit and enroll participants for genetic studies of AD in PR. While genetic studies of AD in PR have been undertaken by several groups as part of a larger emphasis on understanding AD in Caribbean Hispanics (Lee et al., 2006; Barral et al., 2015), the ascertainment approach developed for PRADI focuses solely on the island and intends to create a program that enhances knowledge of AD in PR.

Efforts to increase knowledge of AD in PR have grown recent years and the multisource approach to recruitment and enrollment is aligned with programs such as the *Un Café por el Alzheimer* program in PR, which provides education about AD at coffee shops and through social media (Friedman et al., 2016). The educational component that we include as part of our larger ascertainment approach is crucial for providing information about AD to healthcare providers and the public across the various communities and will potentially impact participation in biomedical research, including genetic studies (Karlawish et al., 2011).

The goal of the PRADI study is to investigate the genetics of AD in Puerto Ricans. AD is a complex disease with substantial burden on the population – particularly in PR where there is a large aging population suffering from chronic diseases that may exacerbate existing risk (Perreira et al., 2017). To date, there have been a scarcity of genetic studies of complex traits (e.g., AD) in Puerto Ricans which could exacerbate existing health disparities. Exceptions to this are the Boston Puerto Rican Health Study (BPRHS), a longitudinal cohort study which examines non-genetic, and genetic influences on multiple health outcomes among mainland Puerto Ricans (Tucker et al., 2010) and the Hispanic Community Health Study (HCHS), a large longitudinal multi-cohort project which studies a variety of health outcomes among different Hispanic-Latino groups in the US, including Puerto Ricans (Lavange et al., 2010) – both of which have extensive phenotypic and genotypic data. Using data from these cohorts, investigators have found links between select genes, obesity and asthma (Guo et al., 2018), lipid profiles (Graff et al., 2017), and blood pressure traits (Sofer et al., 2017). A large amount of research has genetic factors contributing to asthma and other pulmonary traits which are a major health problem in Puerto Ricans. The involvement of Puerto Ricans in this work can lead to greater understanding of genetic contributions to disease in this population and intervention opportunities. Central to the success of this research is ensuring participation in this research (Karlawish et al., 2011).

Our results suggest the importance of engaging multiple stakeholders and communities across municipalities. Including stakeholders in the development of outreach and recruitment was an important part of the PRADI ascertainment approach. Another important aspect of our ascertainment approach was the provision of AD and dementia information to providers, care centers, and the public. While our ascertainment results cannot be directly attributed to our multisource approach we have preliminary data that can guide more systematic evaluation of what works best as the PRADI study moves forward. Ultimately, this study and others like it are intended to inform and improve health outcomes and reduce health disparities for Puerto Ricans and other Hispanic Latino populations who have been consistently underserved.

## Ethics Statement

This study was carried out in accordance to the recommendations of the National Institute of Health Guiding Principles for Ethical Research Pursuing Potential Research Participants Protection and the 2016 National Institute of Health Single Review Board (sIRB) Policy. This study received ethical approval from University of Miami Institutional Review Board (approved protocol #20070307) and Universidad Central del Caribe Institutional Review Board (approved protocol # 2016-26). The Universidad Central del Caribe is relying on the designated UM-IRB by an Institutional Review Board Authorization Agreement (Protocol: Genetic Studies in Dementia). All subjects (participant or proxy) gave written informed consent. This study was carried out in accordance with the Declaration of Helsinki and amendments.

## Author Contributions

MC helped with study design, assisted with clinical adjudication of patient and control data, and wrote and proofread the manuscript. BF-A and KC assisted with study design, ascertainment, and clinical adjudication of patient and control data, and wrote and proofread the manuscript. JR and FR performed statistical analyses and helped to writing the manuscript. LA and JV helped with study design, ascertainment, and clinical adjudication of patient and control data. PB, PRM, AR, and VR helped with ascertainment and clinical adjudication of patient and control data. CS, PM, AG, MP, and JM helped with ascertainment of patient and control data. KH-N compiled data for the publication and ran clinical queries. NF helped with ascertainment of patient and control data, and proofread the manuscript. AC and HA helped with ascertainment of patient and control data, diagnosis, and adjudication. GB conceived of and implemented the study design. MP-V conceived of and implemented the study design, assisted with ascertainment and clinical adjudication of patient and control data, and helped to writing the manuscript.

## Conflict of Interest Statement

The authors declare that the research was conducted in the absence of any commercial or financial relationships that could be construed as a potential conflict of interest.

## Footnotes

1 http://www.salud.gov.pr/Pages/Regiones-de-Salud-y-Servicios-Directos.aspx
